# Correlation between promoter methylation of *p14^ARF^*, *TMS1/ASC*, and *DAPK*, and *p53 *mutation with prognosis in cholangiocarcinoma

**DOI:** 10.1186/1477-7819-10-5

**Published:** 2012-01-09

**Authors:** Liu Xiaofang, Tang Kun, Yu Shaoping, Wang Zaiqiu, Su Hailong

**Affiliations:** 1 Department of Hepatobiliary Surgery, Affiliated Yantai Yuhuangding Hospital, Qingdao University Medical College, Yantai 264000, China

**Keywords:** cholangiocarcinoma, methylation-specific PCR, p53-Bax mitochondrial apoptosis pathway

## Abstract

**Background:**

To study the methylation status of genes that play a role in the p53-Bax mitochondrial apoptosis pathway and its clinical significance in cholangiocarcinoma.

**Patients and Methods:**

Out of 36 cases cholangiocarcinoma patients from April 2000 to May 2005 were collected.Promoter hypermethylation of *DAPK*, *p14^ARF^*, and *ASC *were detected by methylation-specific PCR on cholangiocarcinoma and normal adjacent tissues samples. Mutation of the p53 gene was examined by automated sequencing. Correlation between methylation of these genes and/or *p53 *mutation status with clinical characteristics of patients was investigated by statistical analysis.

**Results:**

We found 66.7% of 36 cholangiocarcinoma patients had methylation of at least one of the tumor suppressor genes analyzed. *p53 *gene mutation was found in 22 of 36 patients (61.1%). Combined *p53 *mutation and *DAPK, p14^ARF^, and/or ASC *methylation was detected in 14 cases (38.9%). There were statistically significant differences in the extent of pathologic biology, differentiation, and invasion between patients with combined *p53 *mutation and *DAPK, p14^ARF^, and/or ASC *methylation compared to those without (P < 0.05). The survival rate of patients with combined *DAPK, p14^ARF^, and ASC *methylation and *p53 *mutation was poorer than other patients (*P *< 0.05).

**Conclusion:**

Our study indicates that methylation of *DAPK, p14^ARF^, and ASC *in cholangiocarcinoma is a common event. Furthermore, *p53 *mutation combined with *DAPK, p14^ARF^, and/or ASC *methylation correlates with malignancy and poor prognosis.

## Background

Apoptosis or programmed cell death is a normal physiological control mechanism that is critical in maintaining homeostasis. Apoptotic inhibition can lead to abnormal cell survival and carcinogenesis. The p53-Bax mitochondrial apoptosis pathway plays an important role in inducing cell death after DNA damage or under conditions of cellular stress [1]. However, some genes in the pathway have latent methylation sites, such as *p14^ARF^*, *DAPK*, and *ASC/TMS1*, which can be methylated and inactivated resulting in cancer development [2]. Studies investigating methylation of gene that play a role in activation of the p53-Bax mitochondrial apoptosis pathway has not been well studied in cholangiocarcinoma to date. Thus, investigation of the methylation status of the genes in this pathway may give insight into the mechanism of cholangiocarcinoma development, resulting in enhanced diagnostic and treatment capabilities. We determined the methylation status of *p14^ARF^*, *DAPK*, and *ASC/TMS1 *as well as *p53 *mutation status in cancerous and normal adjacent tissue for 36 cholangiocarcinoma patients. Correlation between *p14^ARF^*, *DAPK*, and *ASC/TMS1 *methylation and/or *p53 *mutation with the biological behavior of cholangiocarcinoma and clinical outcome was examined.

## Methods

### 1. Patients

Cholangiocarcinoma and normal adjacent tissues samples were randomly selected from specimens obtained and immediately frozen from patients treated by surgical resection from April 2000 to May 2005 in the Department of General Surgery of the First Affiliated Hospital of China Medical University and Hepatobiliary Surgery of the Affiliated Yantai Yuhuangding Hospital of Qingdao University Medical College. Informed consent was obtained from all the patients. Samples included 18 tubular adenocarcinomas, 9 papillary adenocarcinomas, 4 mucoid carcinomas, and 5 non-differentiated carcinomas. Twenty-five samples were well-moderately differentiated and 11 samples were poorly differentiated. According to the UICC standard, 16 cases were T1, 10 cases were T2, and 10 cases were T3. Lymph node metastasis was found in 33 cases. Non-lymph node metastasis was found in the 3 cases. This group contained 23 males and 13 females, with ages ranging from 36 to 71 years (median age of 61.2 years). A11 of the samples were frozen at -80°C until DNA extraction and histological diagnosis by a pathologist.

### 2. DNA Extraction

DNA was obtained from samples using the QIAamp DNA Micro kit according to the manufacturer's instructions (Qiagen, Hilden, Germany). Briefly, tissue sample weighing less than 10 mg was injected into a 1.5 ml microcentrifuge tube, 180 μl Buffer ATL and 20 μl proteinase k was immediately added followed by pulse-vortexing for 15 s. Samples were incubated overnight at 56°C. Buffer AL (200 μl) and 100% ethanol (200 μl) were added and samples incubated for 5 min at room temperature. Lysate was then added to a QIAamp MinElute Column, 500 μl Buffer AW1 and Buffer AW2 added, and samples were centrifuged at 15000 rpm. Buffer AE (100 μl) was added and DNA obtained. DNA was quantified by spectrophotometric absorption and the A260/280 ratio.

### 3. Methylation specific PCR

We examined the methylation status of genes by using the methylation specific polymerase chain reaction (MSP) method. Briefly, 1 mg of tissue DNA was urea/bisulfite-treated to render unmethylated cytosines to uracil according to the method previously described [3] to render unmethylated cytosines to uracil. The modified DNA was resuspended in 20 μl of TE Buffer and immediately subjected to PCR or stored at -20°C. Table 1 shows primer sequences used for PCR. Primers were synthesized by Hokkaido Bioscience Company (Ohtsu, Japan).

**Table 1:** MSP primers sequences

gene	primer sequences	product (bp)
***p***14^ARF^	M: GTG TTA AAG GGCTGGCCGTAGGC	122
	AAA ACC CTC ACT CGC GAC GA	
	U: TTT TTG GTG TTA AAG GGT GGT GTA GT	132
	CAC AAA AAC CCT CAC TCA CAA CAA	
DAPK	M: GGA TAG TCG GAT CGA GTT AAC GTC	98
	CCC TCC CAA ACG CCG A	
	U: GGA GGA TAG TTG GAT TGA GTT AAT GTT	106
	CAA ATC CCT CCC AAA CAC CAA	
TMS1/ASC	M: TTG TAG CGG GGT GAG CGG C	191
	AAC GTC CAT AAA CAA CGC G	
	U: GGT TGT AGT GGG GTG AGT GGT	196
	CAA AAC ATC CAT AAA CAA CAA CAC A	

M: Methylated specific PCR primers. U: Unmethylated specific PCR primers.

PCR reactions were performed with a 20 μl reaction volume containing 1 U of Hot start EX Taq DNA polymerase (Takara Biochemical, Japan), 2 μl of 10X EXtaq Buffer, 2 μl of dNTP mixture, forward and reverse primers (8 pmol each), and 1 μl of DNA template. The conditions for the first PCR reaction were as follows: 96°C for 3 min, 40 cycles: 96°C for 30 s, 60°C for 30 s, 72°C for 30 s; 72°C for 4 min. Intergen (IVD) was used as positive control for methylation. DNA from normal lymphocytes was used as a negative control for methylation, and water was used as a negative control. PCR product (5 μl) was resolved by 1.0% TBE gel electrophoresis. Each MSP reaction was repeated at least three times.

### 4. PCR amplification and DNA sequencing

Primers for amplification of p53 exon 5-8 were synthesized by Hokkaido Bioscience Company(Japan)[4]. PCR used a 20 μL reaction volume containing 1 unit of Hot start EXTaq DNA polymerase (Takara, Biochemical, Japan), 2 μL of 10 × EXTaq Buffer,2 μL of dNTP mixture and each primers (8 pmol each for reaction)and 1 μL of DNA template. The condition of the first PCR was as follows: 96°C 3 min for denaturation, 40 cycles of PCR,(96°C,30s,60°C,30s,72°C,30s) with a final elongation step of 4 min at 72°C.Water was used as negative control. PCR product (5 μl) was resolved by 10 g/L TBE gel electrophoresis. Each PCR reaction was repeated three times.

All of the PCR products were purified using Auto seq TMG-50 (Amersham Biochemical Company, Piscataway, NJ). BigDye Terminator Cycle sequencing Ready Reaction (Perkin Elmer, Waltham, MA) was performed to sequence DNA. The sequencing primers used were the same as the PCR primers, but, the concentration was 1/10th of that used for the PCR reaction. The sequencing reaction condition was as follows: 95°C, 4 min; 40 cycles: 95°C for 30 s, 55°C for 30 s, 72°C for 30 s; 72°C for 4 min. Both sense and antisense products were analyzed on an ABI prism 310 Genetic Analyzer (Perkin Elmer). Each reaction was repeated three times.

### 5. Follow-up data

A follow-up visit at the hospital was offered every three months for the first year after surgery, every six months for the second year, and every six to nine months for the third year. All follow-up data was recorded.

### 6. Statistical analysis

All data were analyzed by Chi-square test or Spearman rank correlation analysis. The survival rate was calculated using the Kaplan-Meier method and compared with long-rank test using SPSS 13.0 software (IBM, Armonk, NY; size of test α = 0.05).

## Results

### 1.*p14^ARF^*, *DAPK*, and *ASC/TMS1 *methylation status in cholangiocarcinoma

**Figure 1:**
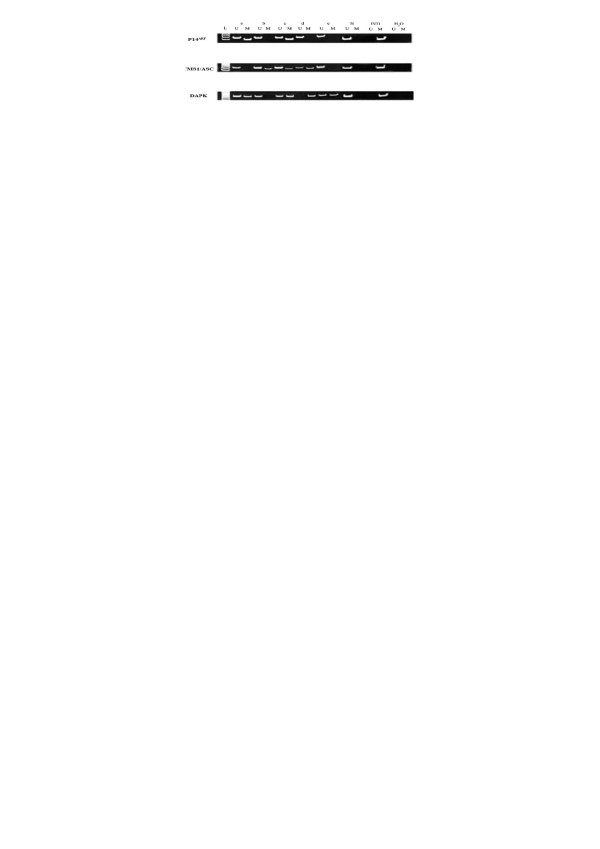
**Methylation specific PCR analysis of *p14^ARF^*, *TMS1/ASC*, and *DAPK *in cholangiocarcinoma and normal adjacent tissue**. L: 100 bp DNA ladder; U: unmethylated primers; M: methylated primers;. a-e:cholangiocarcinoma samples; IVD: positive control; N: negative control.

The methylation status of tumor suppressor genes that play a role in the p53-Bax mitochondrial apoptosis pathway (*p14^ARF^, DAPK, and TMS1/ASC*) was investigated in cholangiocarcinoma samples. It was found that 66.7 of 36 cases had methylation of at least one of these genes. The frequency of methylation in cholangiocarcinoma tissue was: p14^ARF ^(24.0%), DAPK (30.6%), and TMS1/ASC (36.1%). The frequency of methylation in normal adjacent tissue was low: DAPK (5.6%) and TMS1/ASC (8.3%) (Figure 1). Methylation of all tumor suppressor genes analyzed was significantly increased in cholangiocarcinoma samples compared to normal adjacent tissue (P < 0.001).

### 2. *p53 *gene mutations in cholangiocarcinoma tissue

**Figure 2:**
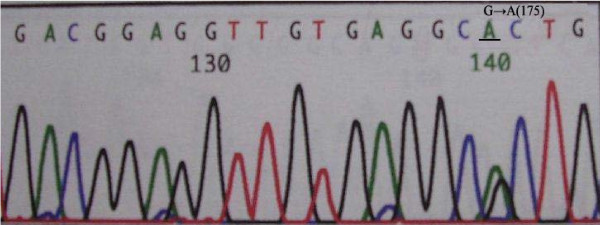
**Representative transition of p53 exon 5 (G: C/A: T) found in cholangiocarcinoma**.

**Figure 3:**
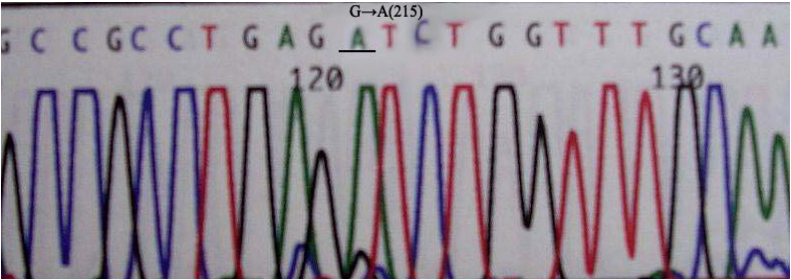
**Representative transition of p53 exon 6 (G: C/A: T) found in cholangiocarcinoma**.

**Figure 4:**
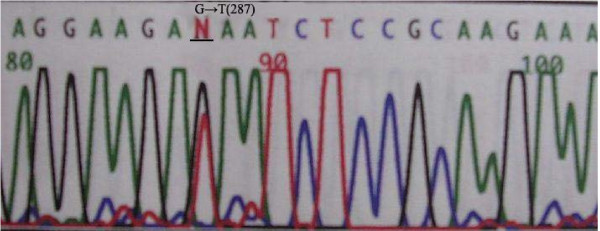
**Representative tranversion of p53 exon 8 (G → T) found in cholangiocarcinoma**.

Exon 5-8 of *p53 *was amplified by PCR in all samples; gene mutations were detected by DNA sequencing. Mutations in *p53 *were detected in 22 of 36 patients (61.1%). There were 7 cases of exon 5 mutations, located on codons 161, 175 and 196 codon. All these mutations were transitions (G:C/A:T). Exon 6 mutations were detected in 6 cases, located on codons 209, 213, and 215; 4 mutations were transitions (G:C/A:T) and 2 mutations were tranversions (G-T). Exon 7 mutations were found in 3 cases, located on codons 248 and 252. Exon 8 mutations were detected in 6 cases, located on codons 282, 287, 289, and 306; 4 mutations were transitions (G:C/A:T) and 2 mutations were tranversions (G-T) (Figure 2, 3, and 4).

### 3. Methylation patterns of *DAPK*, *p14^ARF^*, and *ASC *in cholangiocarcinoma

**Figure 5:**
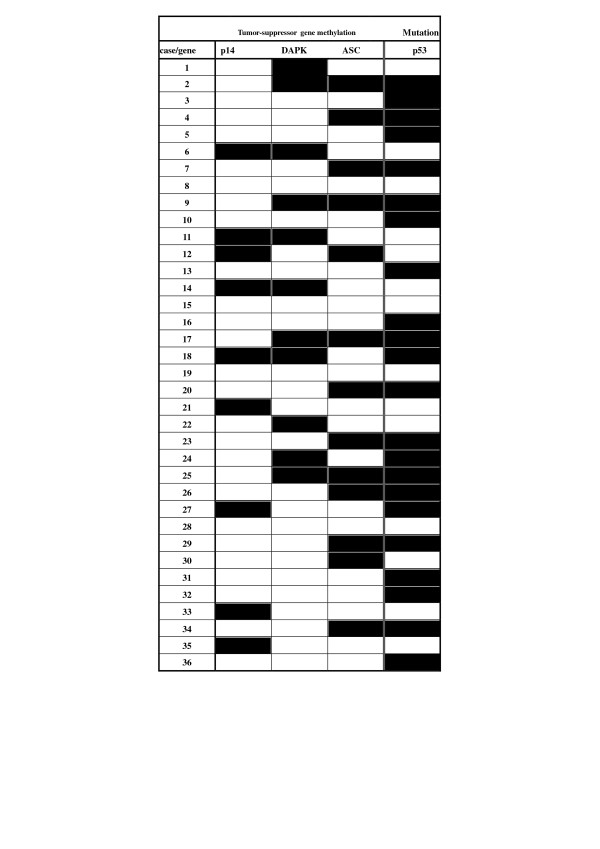
**Methylation profile of *p14^ARF^*, *TMS1/ASC*, and *DAPK *and *p53 *mutation in cholangiocarcinoma**. Open boxes denote no detectable methylation or mutation. Shaded boxes represent positive methylation or mutation.

Promoter region methylation of *p14^ARF^, DAPK, and TMS1/ASC *in cholangiocarcinoma is common. There were 15 cases (41.1%) with methylation of one of these genes, 9 cases (25.0%) with methylation of two of these genes, and no cases where all three genes were methylated. In some cases (13.9%) of one of these genes was found to be methylated in normal adjacent tissue, however, there were no cases where two of these genes were found methylated in normal tissue. None of these genes was found to be methylated at a significantly higher level in tumor tissue than the other genes (P > 0.05). The methylation profile for *p14^ARF^, DAPK, and TMS1/ASC *in all 36 cases of cholangiocarcinoma examined is summarized in Figure 5.

### 4. Correlation between *p14^ARF^, DAPK, and TMS1/ASC *methylation, *p53 *mutation, and pathologic biology in cholangiocarcinoma

There were no statistically significant differences in age or gender in the methylation of *p14^ARF^, DAPK, and TMS1/ASC *or *p53 *gene mutation on p53-Bax mitochondrial apoptosis pathway. Combined *p53 *mutation and *p14^ARF^, DAPK, and/or TMS1/ASC *methylation was found in 14 out of 36 cases (38.9%). Interestingly, *p53 *mutation and *p14^ARF^, DAPK, and/or TMS1/ASC *methylation was often found in mucoid carcinomas and non-differentiated carcinomas, these tumors tended to be poorly differentiated and had a deeper extent of invasion. There was a correlation between the occurrence of *p53 *mutations and tumor pathology, differentiation, and invasion (P < 0.05) (Table 2). Although combined *p53 *gene mutation and *p14^ARF^, DAPK, and/or TMS1/ASC *methylation was often found in patients with lymph node metastasis, this observation was not statistically significant (P > 0.05).

**Table 2:** Correlation between combined *p53 *mutation and *p14^ARF^*, *DAPK*, and/or *ASC/TMS1 *methylation with pathologic biology in cholangiocarcinoma

		combined *p53 *mutation and *p14^ARF^, DAPK*, and/or *ASC/TMS1 *methylation		
**Pathologic character**	**cases**	**positive**	**positive ratio (%)**	**P-value**

**Pathologic type**				
Tubular carcinoma	18	5	27.8	
Papillary carcinoma	9	1	11.1	
Mucoid carcinoma	4	4	100.0	
Non-differentiated	5	4	80.0	< 0.05
carcinoma				
**Differentiation**				
Well -Moderate	25	6	24.0	
Poor	11	8	72.7	< 0.05
**Invasion**				
T1	16	2	12.5	
T2	10	4	40.0	
T3	10	8	80.0	< 0.05
**Lymph node metastasis**				
N0	3	0	0	
N1	33	14	42.4	> 0.05

### 5. Correlation of combined *p14^ARF^, DAPK, and TMS1/ASC *methylation and *p53 *mutation with prognosis in cholangiocarcinoma

**Figure 6:**
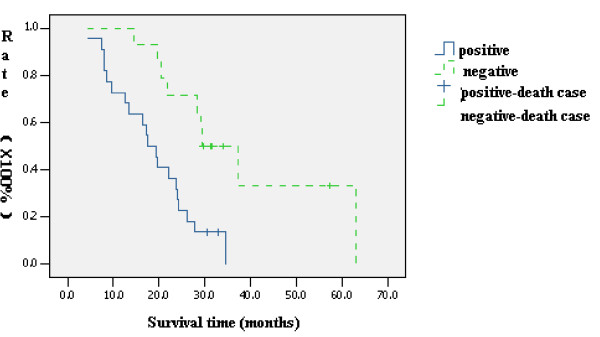
**Kaplan-Meier curve of *p14^ARF^*, *TMS1/ASC*, and *DAPK *methylation and *p53 *mutation on p53-Bax mitochondrial apoptosis pathway and prognosis in cholangiocarcinoma**. Positive: patients with combined *p53 *mutation and *p14^ARF^*, *TMS1/ASC*, and/or *DAPK*; negative: patients without combined *p53 *mutation and *p14^ARF^*, *TMS1/ASC*, and/or *DAPK*; + = a patient death.

Complete follow-up information was available for all the 36 patients enrolled in this study with a mean follow-up time period of 23.78 months, in which 29 patients died and 7 survived. The one-, two-, and three-year survival rates were 44.0%, 21.0%, and 14.0%, respectively, with a median survival time of 22.29 months. The one-, two-, and three-year survival rates of patients with combined methylation of *p14^ARF^, DAPK, and/or TMS1/ASC *and *p53 *mutation was 28.0%, 5.0%, and 0.0%, respectively, with a median survival time of 18.6 months. The one-, two-, and three-year survival rates of patients without combined methylation of the tumor suppressor genes analyzed was 70.0%, 43.0%, and 28.0%, with a median survival time of 33.1 months. The survival rate between the two groups was significantly different (*X^2 ^= *9.06, P < 0.05) (Figure 6). Combined *p53 *mutation and *p14^ARF^, DAPK, and/or TMS1/ASC *methylation correlated with poor prognosis.

## Discussion

Cholangiocarcinoma is a malignant tumor of the epithelial bile duct, and accounts for 3.0% of malignant tumors of the gastrointestinal tract [5,6]. In recent years, the incidence of cholangiocarcinoma and the mortality rate due to this disease have been increasing [7,8]. The mean age of patients with cholangiocarcinoma is 50 years, and the incidence in males is 1.5 times greater than that in females [9-11]. Without special clinical manifestations and tumor markers, early diagnosis and treatment of cholangiocarcinoma is very difficult.

Mammalian cells possess the capacity to epigenetically modify their genomes via the covalent addition of a methyl group to the 5-position of the cytosine ring within the context of the CpG dinucleotide. Certain regions of the genome, which are often clustered at the 5'-ends of genes possess the expected CpG frequency and have been termed CpG islands. CpG island methylation has been shown to be essential for normal development, X-chromosome inactivation, imprinting, and the suppression of parasitic DNA sequences. However, hypermethylation of CpG islands can cause inactivation of genes [12,13].

In this study, promoter hypermethylation of *DAPK*, *p14^ARF^*, and *ASC *was detected by methylation-specific PCR, and mutation of the *p53 *gene status at exons 5-8 was examined by automated sequencing in 36 cases of cholangiocarcinoma. We found that 66.7% of patients had methylation of at least one of the tumor suppressor genes analyzed. The frequency of methylation in cholangiocarcinoma was: p14^ARF ^(24.0%), DAPK (30.6%), and TMS1/ASC (36.1%). The frequency of methylation in normal adjacent tissues was: DAPK (5.6%) and TMS1/ASC (8.3%). *p53 *gene mutations were found in 22 of 36 patients (61.1%). Combined p53 mutation and *DAPK*, *p14^ARF^*, and/or *ASC *methylation was detected in 14 cases (38.9%).

The p53-Bax mitochondrial apoptosis pathway plays an important anti-tumor role. When cells suffer DNA damage or certain types of stress, p53 is activated and sends a death signal to cells. p53 activity is regulated by MDM2, which induces the degradation of p53. MDM2 levels are regulated by p14^ARF^. DNA damage or cellular stress induces expression of p14^ARF^, resulting in degradation of MDM2 and stabilization of p53. Stress signals induce p14^ARF ^expression, in part, through death-associated protein kinase, DAPK. Activated p53 promotes apoptosis through Bax, a molecule that stimulates the release of cytochrome-c from mitochondria, activating procaspase-9 and resulting in cell death. TMS1/ASC plays a role in caspase-9-mediated apoptosis. Expression of regulators of the p53-Bax mitochondrial apoptosis pathway, including p14^ARF^, DAPK, and ASC/TMS1, can become inactivated by promoter methylation and lead to the destruction of the pathway [14,15]. Our study indicates that methylation of regulators of the p53-Bax mitochondrial apoptosis pathway is a common epigenetic event in cholangiocarcinoma.

Futhermore, we found *p53 *gene mutation combined with *DAPK*, *p14^ARF^*, and/or *ASC *methylation in 14 cases (38.9%), which correlated with extent of pathologic biology, differentiation, and invasion of the tumors (P < 0.05). Patients with combined *p53 *mutation and methylation of tumor suppressor genes tended to have more malignant tumors. Moreover, the one-, two-, and three-year survival rate of patients with combined tumor suppressor gene methylation and *p53 *mutation was 28.0%, 5.0%, and 0.0%, respectively, with a median survival time of 18.6 months. Those patients without combined *p53 *mutation and tumor suppressor methylation was 70.0%, 43.0%, and 28.0%, with a median survival of 33.1 months. Therefore, combined *p53 *mutation and *DAPK*, *p14^ARF^*, and/or *ASC *methylation correlates with poor prognosis in cholangiocarcinoma patients.

Although surgery can relieve obstruction of the biliary tract, the distinct pathobiology of cholangiocarcinoma makes it difficult to obtain a radical surgical cure. Thus, chemotherapeutics are an important part of combined therapy. However, at present, it is unclear how effective chemotherapeutics are in treating cholangiocarcinoma patients. Our study indicates the methylation status of regulators of the p53-Bax mitochondrial apoptosis pathway could indicate the biological behavior of cholangiocarcinoma and predict prognosis of these patients, providing a basis for postoperative adjunctive treatment [16]. Further research is needed to investigate the methylation status of p53-Bax mitochondrial apoptosis pathway genes in cholangiocarcinoma patients and how this correlates with response to drug therapy.

## Conclusion

In conclusion, 66.7% of 36 cholangiocarcinoma cases had methylation of at least one of the tumor suppressor genes analyzed. These data indicate that methylation of genes that regulate the p53-Bax mitochondrial apoptosis pathway is a common event cholangiocarcinoma. Mutation of the *p53 *gene was found in 61.1% of cases. Combined *p53 *mutation and *DAPK*, *p14^ARF^*, and/or *ASC *methylation was found in 38.9% of cases, and these patients had a poorer pathologic biology and prognosis than the other patients. Future studies are required to assess *DAPK*, *p14^ARF^*, and *ASC *methylation status and to the possibility of using DNA demethylating drugs to treat cholangiocarcinoma patients.

## Funding

The study was supported by a grant from Natural Science Foundation of Shandong Province, China (No. Y2008C82).

## Competing interests

No benefits in any form have been received or will be received from a commercial party related directly or indirectly to the subject of this article.

## Authors' contributions

LXF proposed the study and wrote the first draft of the manuscript. TK analyzed the data. All authors contributed to the design and interpretation of the study and to further drafts. LXF is the guarantor. All authors read and approved the final manuscript'
